# Herpin equivalence in temporal metamaterials

**DOI:** 10.1515/nanoph-2022-0338

**Published:** 2022-09-08

**Authors:** Giuseppe Castaldi, Massimo Moccia, Nader Engheta, Vincenzo Galdi

**Affiliations:** Department of Engineering, Fields & Waves Lab, University of Sannio, Benevento, I-82100, Italy; Department of Electrical and Systems Engineering, University of Pennsylvania, Philadelphia, PA, 19104, USA

**Keywords:** filters, Herpin equivalence, metamaterials, time-varying

## Abstract

In analogy with spatial multilayers, we put forward the idea of Herpin equivalence in temporal metamaterials characterized by step-like time variations of the constitutive parameters. We show that, at a given frequency, an arbitrary temporal multistep exhibiting mirror symmetry can be replaced by an equivalent temporal slab with suitable refractive index and travel-time. This enables the synthesis of arbitrary values of the refractive index, in a way that differs fundamentally from the effective-medium approach, and adds new useful analytical machinery to the available toolbox for the study and design of temporal metamaterials, with potentially intriguing applications to anti-reflection coatings and filters.

## 1 Introduction

In optics, a generic dielectric multilayer exhibiting mirror symmetry can be treated, for fixed frequency and normal incidence, as a *single* equivalent layer with suitable refractive index and thickness. This mathematical result, usually known as Herpin equivalence [1], enables to exploit a limited number of material constituents (e.g., two) for synthesizing an arbitrary refractive index, subject to the aforementioned limitations, and provides a very powerful analytical tool for the study and design of thin-film anti-reflection coatings and filters [2–4].

Here, we explore to what extent the above concept can be translated to *time-varying* scenarios. Our study is motivated by the mounting interest in *temporal* metamaterials [5], characterized by time modulations of the constitutive parameters, which promise to become technologically viable thanks to the growing availability of rapidly reconfigurable meta-atoms [6]. Within the emerging broad framework of “space-time” metamaterials [7, 8], this has revamped the study of wave interactions with time-varying media – a subject of longstanding interest in electromagnetics [9–11]. By relying on space-time duality, many examples of temporal analogs of canonical problems, effects and concepts that are well-known in spatially variant scenarios have been recently put forward, including temporal boundaries [12] and slabs [13, 14], effective-medium theory [15–17], diffraction gratings [18, 19], anti-reflection coatings [20, 21], tapered lines [22], filters [23], Faraday rotation [24], and Brewster angle [25] (see also Ref. [26] for a recent comprehensive review).

In what follows, we apply the Herpin-equivalence concept to temporal multisteps, characterized by abrupt changes of the refractive index in time, highlighting similarities and differences with respect to the spatial counterpart, and illustrating possible applications to the synthesis of temporal anti-reflection coatings and filters.

## 2 Results and discussion

### 2.1 Problem schematic and statement

**Figure 1:**
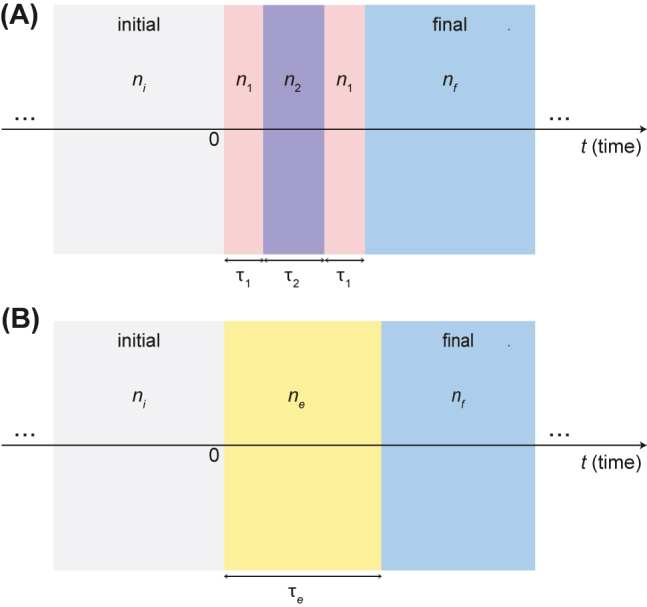
Problem illustration: (A) temporal three-step, and (B) Herpin-equivalent temporal slab (details in the text).

We consider the scenario illustrated in Figure 1(A), featuring a homogeneous, isotropic, non-magnetic (i.e., relative permittivity μ_r_ = 1) medium, subject to a temporal modulation of the dielectric permittivity. This results in a time-varying refractive index which, from an initial (stationary) value *n*_*i*_, starting at a given time instant (chosen as *t* = 0), undergoes four abrupt changes among values *n*_1_, *n*_2_, *n*_1_ (within intervals of duration *τ*_1_, *τ*_2_, and *τ*_1_, respectively), and finally *n*_*f*_ (which is maintained indefinitely). This prototypical example represents the temporal analog of a symmetrical dielectric three-layer sandwiched between two materials (e.g., a substrate and air), and constitutes the elementary brick to treat arbitrary temporal multistep profiles exhibiting mirror symmetry. As in previous studies on temporal multi-steps [15], we assume negligible temporal dispersion and discontinuous transitions in the refractive index; these idealized assumptions are reasonable approximations as long as the operational frequency is far away from any material resonances and the rise/fall times are much smaller than the modulation intervals and wave period, respectively.

Our aim is to show that, at a given frequency and for observation times *t* > 2*τ*_1_ + *τ*_2_, the temporal three-step in Figure 1(A) can be effectively replaced by an equivalent temporal slab with suitable refractive index and duration.

### 2.2 Extension of Herpin equivalence

In the spatial case, Herpin equivalence stems from specific mathematical properties of the transfer-matrix of symmetrical multilayers [1]. Interestingly, the transfer-matrix formalism can be extended to temporal multisteps [27, 28]. Specifically, by assuming a plane-wave excitation with time-harmonic $\exp\left(-i\omega t\right)$ dependence, as detailed in the Methods Section 4.1, the (normalized) electric and magnetic inductions at the beginning and end of each of the modulation intervals in Figure 1(A) are related by a transfer-matrix

(1) $${\underline{\underline{S}}}^{\left(\nu \right)}\left(\varphi \right)=\left[\begin{array}{cc} \cos\varphi_{\nu } & \frac{in_{i}}{n_{\nu }}\sin\varphi_{\nu }\\ \frac{in_{\nu }}{n_{i}}\sin\varphi_{\nu } & \cos\varphi_{\nu } \end{array}\right],\;\nu =1,2,$$

with *φ*_*ν*_ = *ωn*_*i*_*τ*_*ν*_/*n*_*ν*_ denoting normalized travel-times, and *ω* the angular frequency in the initial medium. Remarkably, the transfer-matrix structure in Equation (1) is formally identical with the one encountered in the spatial case [4], with the normalized travel-times playing the role of the electrical thicknesses; in particular, the *unimodular* character is preserved. Likewise, the transfer-matrix describing the entire temporal three-step in Figure 1(A) can be obtained by chain-product of the three single-interval transfer-matrices, viz.,

(2) $$\underline{\underline{S}}={\underline{\underline{S}}}^{\left(1\right)}\cdot {\underline{\underline{S}}}^{\left(2\right)}\cdot {\underline{\underline{S}}}^{\left(1\right)}=\left[\begin{array}{cc} s_{11} & is_{12}\\ is_{21} & s_{22} \end{array}\right],$$

where

(3a) $$\begin{array}{ll} s_{11}=s_{22} & =\cos\left(2\varphi_{1}\right)\cos\varphi_{2}\\ & \;-\frac{1}{2}\left(\frac{n_{1}}{n_{2}}+\frac{n_{2}}{n_{1}}\right)\sin\left(2\varphi_{1}\right)\sin\varphi_{2}, \end{array}$$

(3b) $$\begin{array}{ll} s_{12} & =\frac{n_{i}}{n_{1}}\left[\sin\left(2\varphi_{1}\right)\cos\varphi_{2}+\frac{1}{2}\left(\frac{n_{1}}{n_{2}}+\frac{n_{2}}{n_{1}}\right)\cos\left(2\varphi_{1}\right)\right.\sin\varphi_{2}\\ & \;+\left.\frac{1}{2}\left(\frac{n_{1}}{n_{2}}-\frac{n_{2}}{n_{1}}\right)\sin\varphi_{2}\right], \end{array}$$

(3c) $$\begin{array}{ll} s_{21} & =\frac{n_{1}}{n_{i}}\left[\sin\left(2\varphi_{1}\right)\cos\varphi_{2}+\frac{1}{2}\left(\frac{n_{1}}{n_{2}}+\frac{n_{2}}{n_{1}}\right)\cos\left(2\varphi_{1}\right)\right.\sin\varphi_{2}\\ & \;\left.-\frac{1}{2}\left(\frac{n_{1}}{n_{2}}-\frac{n_{2}}{n_{1}}\right)\sin\varphi_{2}\right]. \end{array}$$

Thus, as for the spatial counterpart [1], the determinant is unity (in view of the product of three unimodular matrices) and the two diagonal elements are identical (in view of mirror symmetry of the temporal three-step); these two constraints imply that the three-step transfer-matrix in Equation (2) is completely determined by *only two* parameters. In other words, by comparing the single-interval transfer-matrix in Equation (1) with that in Equation (2), one can define an equivalent refractive index

(4) $$n_{e}=n_{i}\sqrt{\frac{s_{21}}{s_{12}}},$$

and equivalent normalized travel-time

(5) $$\varphi_{e}=\left\{\begin{array}{c} \arccos\left(s_{11}\right),\;\;\;\text{Re}\left(s_{12}\right)>0,\;\\ 2\pi -\arccos\left(s_{11}\right),\;\text{Re}\left(s_{12}\right)<0,\; \end{array}\right.$$

and duration

(6) $$\tau_{e}=\frac{n_{e}\varphi_{e}}{\omega n_{i}},$$

so that the symmetrical temporal three-step in Figure 1(A) can be replaced by a *single equivalent* temporal slab, as schematized in Figure 1(B). This yields the sought extension of Herpin equivalence to temporal scenarios. It is important to stress that, as for the spatial counterpart, the above equivalence is *exact*, but restricted to a *single frequency*, and that there are infinite (periodic) solutions for the equivalent travel-time and duration in Equations (5) and (6).

It can be shown that, in the limit of short intervals *τ*_1,2_ ≪ *T* (with *T* = 2*π*/*ω* denoting the wave period), the above equivalence reduces to conventional effective-medium homogenization [15–17], viz.,

(7a) $$n_{e}∼{\left(\frac{\delta_{1}}{n_{1}^{2}}+\frac{\delta_{2}}{n_{2}^{2}}\right)}^{-\frac{1}{2}},$$

(7b) $$\tau_{e}∼2\tau_{1}+\tau_{2},$$

where *δ*_1_ = 2*τ*_1_/(2*τ*_1_ + *τ*_2_) and *δ*_2_ = *τ*_2_/(2*τ*_1_ + *τ*_2_) = 1 − *δ*_1_ denote the duty cycles, i.e., the temporal equivalent of the filling fractions in spatial multilayers. Nevertheless, we stress that Herpin equivalence is fairly distinct from homogenization, and is not restricted to that specific range of applicability and corresponding bounds. For instance, the equivalent index *n*_*e*_ is not restricted to be an intermediate value between the two constituents’ *n*_1_ and *n*_2_.

As anticipated, the above result can be readily extended to an *arbitrary* mirror-symmetric temporal multistep, by starting from the central three intervals, and applying iteratively the equivalence until the entire profile is replaced by a single equivalent temporal slab.

As for possible extensions to account for material dispersion and losses, from the mathematical viewpoint, the equivalence implied by Equations (4)–(6) is strictly valid at a single frequency, and remains valid for complex-valued refractive indices *n*_1_ and *n*_2_. Therefore, one could envision a potential extension to perturbatively account for weak dispersion and losses. Note that, in this case, both the Herpin-equivalent index *n*_*e*_ and the duration *τ*_*e*_ will be generally complex-valued. While a complex-valued *τ*_*e*_ could still be utilized in the analysis (i.e., replacing a temporal multistep with an equivalent slab), a real-valued duration is clearly necessary for the synthesis, and this may only be obtained for specific combinations of the three-step parameters. However, we highlight that the rigorous modeling of time-varying materials in the presence of dispersion entails additional phenomena that are not captured by the simple transfer-matrix approach utilized here. For instance, it was recently shown that a temporal boundary induced in a Lorentzian-type dispersive medium (by abruptly changing its plasma frequency) gives rise to two shifted frequencies, which require additional boundary conditions [29]. While some type of equivalence may still be worked out, it is clear that the space-time analogy is much less straightforward and meaningful.

For design purposes, in order to find the constitutive and geometrical parameters of a temporal three-step for given values of the Herpin-equivalent index and duration, it may be useful to invert the formulation in Equations (4)–(6) [with Equation (3)]. Given the nonlinear character of the equations involved, the solution cannot be generally found in closed form, and it is evidently not unique (in view of the half-wave periodicity). In what follows, we utilize a numerical approach detailed in the Methods Section 4.2.

### 2.3 Representative results

**Figure 2:**
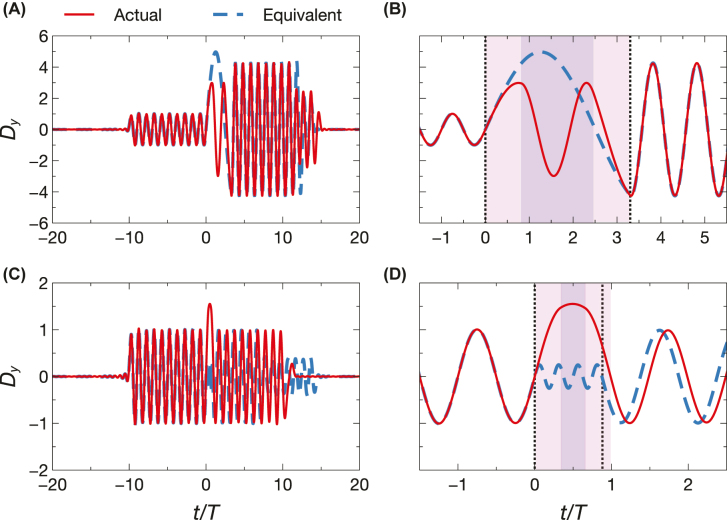
Basic illustration of Herpin equivalence. (A) Numerically computed electric induction (normalized by the incident value $D_{y}^{\left(in\right)}$ ) as a function of (normalized) time, for a symmetrical temporal three-step (*n*_1_ = 3, *n*_2_ = 1.5, *τ*_1_ = 0.823*T*, *τ*_2_ = 1.654*T*; red-solid) and Herpin-equivalent temporal slab (*n*_*e*_ = 5, *τ*_*e*_ = 3.3*T*; blue-dashed); the initial and final indices are *n*_*i*_ = *n*_*f*_ = 1. A windowed sinusoidal plane-wave of period *T* and duration 20*T* is assumed [see Equation (31)], and the observable is sampled at a fixed location *x* = 10*λ*. (B) Magnified detail around *t* = 0. The shaded areas indicate the modulation interval, whereas the vertical dotted lines delimit the Herpin-equivalent duration; in this example, these quantities coincide. (C), (D) Same as panels (A) and (B), respectively, but for temporal three-step with *n*_1_ = 1.5, *n*_2_ = 3, *τ*_1_ = 0.337*T*, *τ*_2_ = 0.319*T*, corresponding to a Herpin-equivalent temporal slab with *n*_*e*_ = 0.25, *τ*_*e*_ = 0.881*T* (see our note about the issue of dispersion of materials with *n*_*e*_ < 1 in the Methods Section 4.3). For this example, the modulation interval does not coincide with the Herpin-equivalent duration (note the temporal shift for *t* > 2*τ*_1_ + *τ*_2_).

For a basic illustration of the equivalence concept, Figure 2(A) compares the response (normalized electric-induction at a fixed location) of a temporal three-step featuring *n*_1_ = 3, *n*_2_ = 1.5, *τ*_1_ = 0.823*T* and *τ*_2_ = 1.654*T*, with that of a Herpin-equivalent temporal slab with *n*_*e*_ = 5 and *τ*_*e*_ = 3.30*T*. These simulations are carried out via a rigorous numerical approach assuming a finite-energy narrowband excitation (see the Methods Section 4.3 for details). Specifically, we assume a windowed sinusoidal plane-wave of period *T* and duration 20*T* [see Equation (31)], and we sample the electric induction at a fixed location *x* = 10*λ* (with *λ* denoting the wavelength corresponding to the period *T*); at the observation point, the source turn-on and turn-off times are *t* = −10*T* and *t* = 10*T*, respectively. In this example, the parameters have been selected (see the Methods Section 4.2 for details) so as to approximately equalize the equivalent and actual durations (i.e., *τ*_*e*_ ≈ 2*τ*_1_ + *τ*_2_), but in principle they could be different. It is also interesting to note that the Herpin-equivalent index is higher than those of the two constituents (*n*_1_ and *n*_2_), which implies that it could not be attained via a mixing formula (assuming positive-permittivity constituents) [15]. As can be observed from the magnified detail around *t* = 0 shown in Figure 2(B), after the modulation interval (i.e., for *t* > 2*τ*_1_ + *τ*_2_) the waveforms are hardly distinguishable (with the exception of late-time effects attributable to the temporal truncation of the source), but they differ substantially inside it; obviously, they also coincide before the modulation interval (*t* < 0), due to causality. Similar considerations also hold for the magnetic induction (not shown for brevity). Thus, at a given frequency, the actual and equivalent profiles yield identical forward and backward waves.

Figure 2(C) and (D) illustrate another representative example, for a temporal three-step featuring *n*_1_ = 1.5, *n*_2_ = 3, *τ*_1_ = 0.337*T*, and *τ*_2_ = 0.319*T*, yielding a Herpin-equivalent temporal slab with *n*_*e*_ = 0.25 and *τ*_*e*_ = 0.881*T*. Interestingly, in this case, the Herpin-equivalent index is smaller than one, unlike both constituents’ (see our note about the issue of dispersion of materials with *n*_*e*_ < 1 in the Methods Section 4.3). Similar considerations hold as for the previous example, but now the Herpin-equivalent duration does not coincide with the physical one (i.e., *τ*_*e*_ ≠ 2*τ*_1_ + *τ*_2_) and, as a consequence, a temporal shift is observed after the modulation interval (i.e., for *t* > 2*τ*_1_ + *τ*_2_).

**Figure 3:**
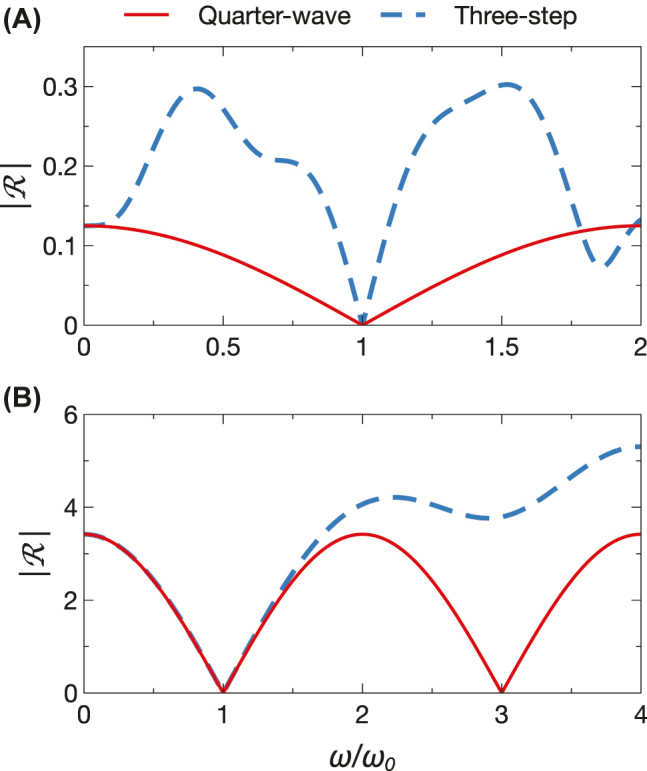
Application to anti-reflection temporal coatings. (A) Example of temporal quarter-wave transformer at a temporal boundary between *n*_*i*_ = 1 and *n*_*f*_ = 2. Temporal reflection coefficient (magnitude) as a function of normalized frequency for an ideal design ( $n_{QW}=\sqrt{2}$ , *τ*_QW_ = 0.354*T*_0_; red-solid) and a three-step (*n*_1_ = 1.5, *n*_2_ = 3, *τ*_1_ = 0.551*T*_0_ and *τ*_2_ = 1.538*T*_0_; blue-dashed) designed so that *n*_*e*_ = *n*_QW_ and *τ*_*e*_ = *τ*_QW_. (B) Same as panel (A), but for a temporal boundary between $n_{i}=\sqrt{10}$ and *n*_*f*_ = 1, yielding an ideal design with *n*_QW_ = 1.778 and *τ*_QW_ = 0.141*T*_0_, and a three-step with *n*_1_ = 1.5, *n*_2_ = 3, *τ*_1_ = 0.049*T*_0_ and *τ*_2_ = 0.035*T*_0_.

As for spatial scenarios, a useful application of Herpin equivalence is the synthesis of an arbitrary refractive index by relying only on a limited set of available values (e.g., two). To illustrate this potential, we recall the recently introduced concept of temporal quarter-wave transformer [20] which, inspired by its spatial counterpart, aims at suppressing the backward wave at a temporal boundary between two refractive-index values *n*_*i*_ and *n*_*f*_, by inserting an intermediate temporal slab with refractive index $n_{QW}=\sqrt{n_{i}n_{f}}$ and duration *τ*_QW_ = *n*_QW_*T*_0_/(4*n*_*i*_), with *T*_0_ = 2*π*/*ω*_0_ denoting the wave period at the desired center angular frequency *ω*_0_ (in the initial medium). Figure 3 illustrates two examples taken from Ref. [20]. Specifically, in Figure 3(A), we assume *n*_*i*_ = 1 and *n*_*f*_ = 2, for which a value $n_{QW}=\sqrt{2}$ is required. In this case, a three-step profile is designed, with *n*_1_ = 1.5, *n*_2_ = 3, *τ*_1_ = 0.551*T*_0_ and *τ*_2_ = 1.538*T*_0_, so that the Herpin-equivalent temporal slab satisfies the sought quarter-wave matching conditions (see the Methods Section 4.2 for details). Note that, in this example, the refractive index to synthesize is lower than those of the two constituents; once again, such value could not be attained via an effective-medium approach [15]. On the other hand, as can be observed, this equivalence is only valid nearby the design frequency, and therefore the operational bandwidth is significantly narrower than that of a conventional quarter-wave transformer. Figure 3(B) shows another example, featuring $n_{i}=\sqrt{10}$ and *n*_*f*_ = 1, for which the required index value *n*_QW_ = 1.778 is synthesized via a three-step with same constituents (*n*_1_ = 1.5 and *n*_2_ = 3) but with *τ*_1_ = 0.049*T*_0_ and *τ*_2_ = 0.035*T*_0_. In this case, the synthesis is less challenging, as the required index value is intermediate between the two constituents’, and the result is not very different from that of a mixing formula. As a consequence, the agreement between the actual and ideal responses remains good over a significantly broader frequency range, and only breaks down at higher frequencies.

As previously mentioned, other typical applications of the conventional (spatial) Herpin equivalence are in the design of thin-film optical filters [2–4]. Before exploring possible translations of these ideas to temporal scenarios, it is important to recall some fundamental differences in the meaning of “reflection” and “transmission”, stemming from causality and lack of power conservation. Specifically, despite the formal analogy between the transfer-matrices, the temporal reflection (backward-wave) and transmission (forward-wave) coefficients do not coincide with their spatial counterparts. As detailed in the Methods Section 4.1, they can be expressed as

(8a) $$R=\frac{n_{i}^{2}\left(s_{22}-is_{21}\right)}{2n_{f}^{2}}-\frac{n_{i}\left(s_{11}-is_{12}\right)}{2n_{f}},$$

(8b) $$T=\frac{n_{i}^{2}\left(s_{22}-is_{21}\right)}{2n_{f}^{2}}+\frac{n_{i}\left(s_{11}-is_{12}\right)}{2n_{f}},$$

from which, recalling the real-valuedness of the coefficients in Equation (3), it can be verified that

(9) $${\left|T\right|}^{2}={\left|R\right|}^{2}+{\left(\frac{n_{i}}{n_{f}}\right)}^{3}.$$

Hence, unlike the spatial case, the temporal transmittance and reflectance are generally not smaller than one and not complementary, but instead they only differ by a constant pedestal. This implies that the conventional “bandpass” and “bandstop” concepts in the spatial case (intended as frequency ranges with low-reflectance/high-transmittance and vice versa, respectively) cannot be translated to the temporal scenario. Nevertheless, some ideas and design tools can still be utilized. In particular, we explore the temporal analogs of the possibly simplest edge filters, based on quarter-wave stacks alternating low- and high-index layers [4]. Accordingly, we consider two basic configurations of temporal multisteps, which can be symbolically represented as

(10a) $$\frac{L}{2}HLHL\ldots LH\frac{L}{2}↔{\left(\frac{L}{2}H\frac{L}{2}\right)}^{N},$$

(10b) $$\frac{H}{2}LHLH\ldots HL\frac{H}{2}↔{\left(\frac{H}{2}L\frac{H}{2}\right)}^{N},$$

where the symbols *L* and *H* denote low and high index, respectively, and the division by two indicates that the duration of the initial and final intervals is halved. As symbolically indicated on the right hand sides of Equation (10), these multisteps can be alternatively viewed as periodic repetitions of *N* symmetrical three-steps, which differ only in the arrangement of the low- and high-index intervals. This implies that the Herpin equivalence can be applied for studying these temporal multisteps. In particular, we choose the travel-times for the low- and high-index intervals so that 2*τ*_1_*n*_*i*_/*n*_1_ = *τ*_2_*n*_*i*_/*n*_2_ = *T*_0_/4, and parameterize the corresponding normalized values

(11) $$2\varphi_{1}=\varphi_{2}=\Omega \frac{\pi }{2}$$

in terms of the normalized frequency Ω = *ω*/*ω*_0_.

For Ω = 2, it can be readily verified that the three-step transfer-matrix in Equation (2) reduces to the identity matrix, irrespective of the arrangements in Equation (10). In this case, a 0/0 indeterminate form is encountered in the Herpin-equivalent index in Equation (4), whose straightforward solution yields

(12) $$n_{e}=n_{1}\sqrt{\frac{n_{1}}{n_{2}}},$$

i.e., two different values for the arrangements in Equation (10). Essentially, these correspond to two half-wave-type conditions which, as already observed in Ref. [23], reproduce the *transparency* condition ( R=0 , T=1 ) encountered in the spatial counterpart [4].

Much less straightforward is the behavior around Ω = 1 (i.e., quarter-wave-type). In this case, the Herpin-equivalent index and related travel-time do not admit real values. Specifically, at Ω = 1, we obtain

(13) $$n_{e}=in_{1},\;\varphi_{e}=\pi -ip\;\text{arcosh}\left[\frac{1}{2}\left(\frac{n_{1}}{n_{2}}+\frac{n_{2}}{n_{1}}\right)\right],$$

where *p* = ±1 for *n*_1_ ≷ *n*_2_. In spatial multilayers [4], this corresponds to an *evanescent* attenuation, which yields high reflection and low transmission. However, as already observed in Ref. [23], this is not the case for the temporal counterpart, where the wavefield may in fact extract power from the system, thereby getting *amplified*. The frequency range wherein this response occurs corresponds to the condition |*s*_11_| > 1, and can therefore be estimated from Equation (3a). For the bilateral bandwidth (centered at Ω = 1), we obtain

(14) $$\Delta \Omega =\frac{4}{\pi }\arcsin\left|\frac{n_{1}-n_{2}}{n_{1}+n_{2}}\right|,$$

which is formally analogous to the bandgap width in the spatial counterpart [4]. The Herpin equivalence provides a powerful tool for the analytical calculation of the temporal reflection and transmission coefficients (at the normalized center frequency Ω = 1) of the multi-steps in Equation (10). As previously mentioned, once the basic three-step has been replaced by an equivalent temporal slab, the entire periodic repetition can be replaced by an equivalent temporal slab of total duration *Nτ*_*e*_. Accordingly, its transfer-matrix is given by Equation (1) with

(15) $$s_{11}=s_{22}=\cos\left(N\varphi_{e}\right),$$

(16) $$s_{12}=p\frac{n_{i}}{n_{e}}\sin\left(N\varphi_{e}\right),\;s_{21}=-p\frac{n_{e}}{n_{i}}\sin\left(N\varphi_{e}\right).$$

From Equation (8a), we then obtain the temporal reflection coefficient

(17) $$R=\frac{n_{i}}{2n_{f}}\left[\left(\frac{n_{i}}{n_{f}}-1\right)\;\cos\left(N\varphi_{e}\right)+p\left(\frac{n_{i}}{n_{1}}+\frac{n_{1}}{n_{f}}\right)\;\sin\left(N\varphi_{e}\right)\right],$$

and, similarly, from Equation (8b), the transmission coefficient (not shown for brevity). Recalling the complex-valued character of *φ*_*e*_, we can obtain a particularly simple and insightful approximation in the asymptotic limit *N* ≫ 1. In particular, assuming *n*_*i*_ = *n*_*f*_ = 1, we obtain for the magnitude:

(18) $$\left|R\right|∼\frac{\left(n_{1}^{2}+1\right)}{4n_{1}}{\left(\frac{n_{H}}{n_{L}}\right)}^{N}.$$

Equation (18) clearly shows that the reflection (backward wave) coefficient is greater than one in magnitude, and grows exponentially with the number of periods, which is consistent with the well-known instability phenomena that can occur in infinite, periodic temporal multi-steps [30]. Interestingly, although this general trend is independent of the arrangements in Equation (10), the multiplying coefficient is different in the two cases.

**Figure 4:**
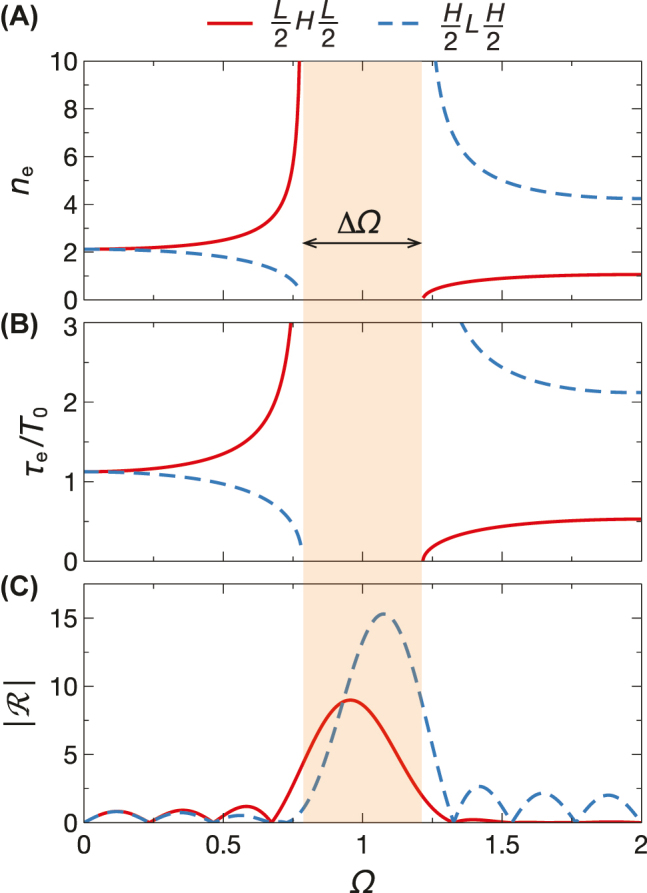
Example of temporal-multistep filters. (A), (B) Herpin-equivalent index and corresponding (normalized) duration, respectively, as a function of normalized frequency, for three-steps with *n*_*L*_ = 1.5, *n*_*H*_ = 3, and *n*_*i*_ = *n*_*f*_ = 1, in the two possible arrangements in Equation (10) (i.e., 0.5*LH*0.5*L*, red-solid; 0.5*HL*0.5*H*, blue-dashed). (C) Corresponding temporal reflection coefficient (magnitude) for *N* = 4 periods. The orange-shaded area indicates the bandgap.

Figure 4 illustrates two representative examples of temporal multistep filters, assuming *n*_*L*_ = 1.5, *n*_*H*_ = 3, *n*_*i*_ = *n*_*f*_ = 1, and *N* = 4. Specifically, Figure 4(A) shows the Herpin-equivalent index, as a function of the normalized frequency, for the two types of three-steps in Equation (10). As can be observed, in the low-frequency limit, for both cases the value approaches the effective-medium-theory prediction [15] in Equation (7a). However, as the frequency grows, the behaviors tend to diverge and, at the lower band-edge Ω = 1 − ΔΩ/2, one branch grows indefinitely [0.5*LH*0.5*L*, i.e., Equation (10a)], whereas the other tends to zero [0.5*HL*0.5*H*, i.e., Equation (10b)]. As previously noted, the values become purely imaginary inside the bandgap and, at the upper band-edge Ω = 1 + ΔΩ/2, the extreme values of the two branches are reversed. Finally, at the half-wave condition (Ω = 2), the values predicted by Equation (12) are observed. As shown in Figure 4(B), the corresponding equivalent durations exhibit qualitatively similar behaviors.

From the temporal reflection coefficients (magnitude) shown in Figure 4(C), we observe the predicted transparency condition at Ω = 2. Moreover, it is worth noticing that the 0.5*HL*0.5*H* configuration in Equation (10b) exhibits a rather flat response, whereas the 0.5*LH*0.5*L* profile in Equation (10a) exhibits a significant ripple. Around Ω = 1, we observe the expected strong reflection peaks in both cases, with different amplitudes. Remarkably, although the number of periods (*N* = 4) is not very high in this example, the simple asymptotic approximation in Equation (18) still provides very accurate estimates (error ≲ 0.5%). Together with Equation (14), it provides a simple analytical approach to control the basic features (in-band reflection and bandwidth) of the filter response.

Qualitatively similar considerations hold for the transmission (forward-wave) coefficient (not shown for brevity), whose intensity essentially differ by a constant pedestal [see Equation (9)].

**Figure 5:**
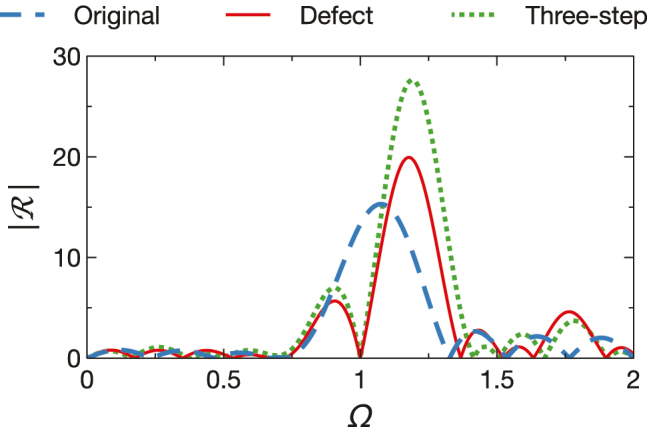
Temporal reflection coefficient (magnitude) for the original 0.5*HL*0.5*H* design in Figure 4 (blue-dashed) compared with the defected filter configuration in Equation (19) (with *n*_*L*_ = 1.5, *n*_*H*_ = 3, 2*τ*_*L*_ = *τ*_*H*_ = *T*_0_/4, *n*_*i*_ = *n*_*f*_ = 1), considering the actual defect interval (*n*_*D*_ = 2.039, *τ*_*D*_ = 1.438*T*_0_; red-solid) and its Herpin-equivalent three-step (*n*_1_ = *n*_*H*_, *n*_2_ = *n*_*L*_, *τ*_1_ = 0.161*T*_0_, *τ*_2_ = 0.877*T*_0_; green-dotted).

As a further example, inspired once again by edge filters [4], we introduce a center “defect” interval in the designs of Figure 4, in order to place a zero at Ω = 1, thereby splitting the reflection peak. Accordingly, with reference to the filter configuration in Equation (10b) (with *N* = 4), we consider the design

(19) $${\left(\frac{H}{2}L\frac{H}{2}\right)}^{2}D{\left(\frac{H}{2}L\frac{H}{2}\right)}^{2},$$

where “*D*” symbolically indicates the defect interval, whose refractive index and duration are chosen by enforcing R=0 at Ω = 1; this yields *n*_*D*_ = 2.039, *τ*_*D*_ = 1.438*T*_0_. In fact, by exploiting Herpin equivalence, this defected interval can be synthesized in terms of a three-step relying on the two original constituents; this yields *n*_1_ = *n*_*H*_, *n*_2_ = *n*_*L*_, *τ*_1_ = 0.161*T*_0_, *τ*_2_ = 0.877*T*_0_. Figure 5 compares the two responses (with the actual defect and Herpin-equivalent three-step) and the original design in Figure 4. We observe the prescribed behavior around Ω = 1, with an asymmetric splitting of the reflection peak.

## 3 Conclusions

In conclusion, we have extended the concept of Herpin equivalence to temporal metamaterials, illustrating similarities and differences with respect to its spatial counterpart. As for spatial scenarios, Herpin equivalence allows to synthesize arbitrary values of the refractive index (at a given frequency), and provides a powerful analytical tool for the study of temporal multisteps, which can find useful applications to the design of temporal anti-reflection coatings and filters.

As possible extensions, more general analytical expressions for the observables of interest can be derived, not restricted to the quarter- or half-wave cases, along the lines of the spatial counterparts [4]. Also worth of interest are possible extensions to deal with anisotropic constituents [31].

From the implementation viewpoint, our approach implies technological challenges that are common to all temporal metamaterials. In fact, the possibility to surrogate a desired refractive index by means of two (possibly more accessible) constituents may be particularly useful in practice. Similar to previously proposed temporal multisteps [15, 20], possible technologically viable implementations could rely on demonstrated time-varying platforms at microwave [32, 33], terahertz [34], and optical [35] frequencies. In particular, the reader is referred to Ref. [26] for a recent review of experimental results, and to Refs. [36, 37] for more recent experimental demonstrations at microwave frequencies.

## 4 Methods

### 4.1 Analytical modeling

Our analytical modeling of the temporal multisteps (with the corresponding results in Figures 3–5) relies on rigorous transfer-matrix formalism. By assuming, without loss of generality, a *y*-polarized plane-wave propagating along the positive *x* direction in a medium with refractive index *n*_*i*_, and a generic temporal slab with refractive index *n*_*m*_ and temporal boundaries at *t* = *t*_*m*_ and *t* = *t*_*m*_ + *τ*_*m*_, the electric and magnetic inductions can be written as

(20a) $$\begin{array}{ll} D_{y}\left(t\right) & =D_{y}\left(t_{m}\right)\cos\left[\frac{n_{i}\omega \left(t-t_{m}\right)}{n_{m}}\right]-\frac{iB_{z}\left(t_{m}\right)n_{1}}{Z_{0}}\sin\left[\frac{n_{i}\omega \left(t-t_{m}\right)}{n_{m}}\right], \end{array}$$

(20b) $$\begin{array}{ll} B_{z}\left(t\right) & =B_{z}\left(t_{m}\right)\cos\left[\frac{n_{i}\omega \left(t-t_{m}\right)}{n_{m}}\right]-\frac{iZ_{0}D_{y}\left(t_{m}\right)}{n_{m}}\sin\left[\frac{n_{i}\omega \left(t-t_{m}\right)}{n_{m}}\right], \end{array}$$

where *Z*_0_ is the vacuum intrinsic impedance, and a common spatial-dependence term $\exp\left(ikx\right)$ has been omitted (with *k* denoting the conserved momentum). To highlight the formal analogies with the spatial scenario, it is expedient to define some equivalent voltages and currents

(21) $$V↔\frac{cB_{z}}{n_{i}},\;I↔-\frac{D_{y}}{n_{i}^{2}\varepsilon_{0}},$$

with *c* and *ɛ*_0_ denoting the vacuum wavespeed and dielectric permittivity, respectively. Then, from Equation (20), the equivalent voltages and currents at the two temporal boundaries can be related as follows

(22) $$\left[\begin{array}{c} V\;\left(t_{m}+\tau_{m}\right)\\ I\;\left(t_{m}+\tau_{m}\right) \end{array}\right]={\underline{\underline{S}}}^{\left(m\right)}\cdot \left[\begin{array}{c} V\;\left(t_{m}\right)\\ I\;\left(t_{m}\right) \end{array}\right],$$

with the transfer matrices defined as in Equation (1), with *α* = *m*. By applying this process iteratively at the temporal three-step in Figure 1(A), it follows straightforwardly that the final (i.e., at *t* = 2*τ*_1_ + *τ*_2_) and initial (i.e., at *t* = 0) values can be obtained by chain product of the single-interval transfer matrices, as in Equation (2), viz.

(23) $$\left[\begin{array}{c} V_{f}\\ I_{f} \end{array}\right]=\left[\begin{array}{cc} s_{11} & is_{12}\\ is_{21} & s_{22} \end{array}\right]\cdot \left[\begin{array}{c} V_{i}\\ I_{i} \end{array}\right],$$

with the matrix elements given by Equation (3).

Assuming an incident amplitude $D_{y}^{\left(in\right)}=\varepsilon_{0}n_{i}^{2}$ , the total electric and magnetic inductions before the first temporal boundary (*t* = 0) and after the final one (*t* = *t*_*f*_ = 2*τ*_1_ + *τ*_2_) can be expressed as

(24a) $$D_{y}\left(t\right)=\varepsilon_{0}\left\{\begin{array}{c} n_{i}^{2}\exp\left(-i\omega t\right),\;\;\;\;\;\;\;\;\;\;\;\;\;\;\;\;t<0,\;\\ n_{f}^{2}\left\{T\exp\left[-\frac{i\omega n_{i}}{n_{f}}\left(t-t_{f}\right)\right]+\;R\exp\left[\frac{i\omega n_{i}}{n_{f}}\left(t-t_{f}\right)\right]\right\},\;t>t_{f},\; \end{array}\right.$$

(24b) $$B_{z}\left(t\right)=\frac{1}{c}\left\{\begin{array}{c} n_{i}\exp\left(-i\omega t\right),\;\;\;\;\;\;\;\;\;\;\;\;\;\;\;\;t<0,\;\\ n_{f}\left\{T\exp\left[-\frac{i\omega n_{i}}{n_{f}}\left(t-t_{f}\right)\right]-R\exp\left[\frac{i\omega n_{i}}{n_{f}}\left(t-t_{f}\right)\right]\right\},\;t>t_{f},\; \end{array}\right.$$

where R and T denote the temporal reflection (backward-wave) and transmission (forward-wave) coefficients, respectively [12]. By particularizing these expressions at the initial and final temporal boundaries, we obtain via Equation (21)

(25a) $$V_{i}=1,\;I_{i}=-1,$$

(25b) $$V_{f}=\frac{n_{f}}{n_{i}}\left(T-R\right),\;I_{f}=-{\left(\frac{n_{f}}{n_{i}}\right)}^{2}\left(T+R\right).$$

Finally, by substituting Equations (25) in (23), we obtain a linear system in the unknowns R and T , whose solution yields the expressions in Equation (8).

### 4.2 Parameter inversion

For inverting Equations (4)–(6), i.e., retrieving the constitutive and geometrical parameters of a temporal three-step (*n*_1_, *n*_2_, *τ*_1_, *τ*_2_) for given values of the Herpin-equivalent index *n*_*e*_ and duration *τ*_*e*_, we consider the following cost function

(26) $$\begin{array}{ll} J\left(n_{1},n_{2},\tau_{1},\tau_{2}\right) & =\left|n_{i}\sqrt{\frac{s_{21}}{s_{12}}}-n_{e}\right|+\left|s_{11}-\cos\varphi_{e}\right|+\left|s_{12}-\frac{n_{i}}{n_{e}}\sin\varphi_{e}\right|, \end{array}$$

with the transfer-matrix coefficients given by Equation (3). It can be readily verified that this function vanishes when Equations (4)–(6) are exactly satisfied, i.e., when the transfer-matrices of the temporal three-step and the Herpin-equivalent slab coincide. Note that the third term in Equation (26) is necessary to enforce the condition on Re(*s*_12_) in Equation (5). Specifically, in our implementation, we chose the values *n*_1_ and *n*_2_ (e.g., *n*_1_ = 1.5 and *n*_2_ = 3), and calculate the durations *τ*_1_ and *τ*_2_ by minimizing the cost function in Equation (26) via the NMinimize routine available in Mathematica™ [38]. This routine implements, among others, the Nelder–Mead method, which we have found to provide a generally satisfactory convergence. In view of the nonlinear character of the equations involved, for given values of *n*_*e*_ and *τ*_*e*_, some tweaking on the choice of *n*_1_ and *n*_2_ may be necessary in order to find valid solutions for *τ*_1_ and *τ*_2_. Moreover, given the half-wave periodicity involved, when a solution for *τ*_1_ and *τ*_2_ exist, it is generally not unique, and we consider the shortest possible values.

### 4.3 Numerical simulations

The simulations in Figure 2 are carried out via a rigorous numerical approach that was already successfully utilized in previous studies [14, 17]. In essence, assuming an arbitrary time-varying relative permittivity *ɛ*(*t*), we synthesize the electric and magnetic inductions via Fourier transform as

(27a) $$D_{y}\left(x,t\right)=\overset{\infty }{\underset{-\infty }{\int }}d_{y}\left(k,t\right)e^{ikx}dk,$$

(27b) $$B_{z}\left(x,t\right)=\overset{\infty }{\underset{-\infty }{\int }}b_{z}\left(k,t\right)e^{ikx}dk,$$

with $d_{y}\left(k,t\right)$ and $b_{z}\left(k,t\right)$ denoting time-dependent plane-wave spectra. From these latter, we define the auxiliary functions

(28) $$u_{1}\left(k,t\right)=\frac{d_{y}\left(k,t\right)}{D_{0}},\;\;u_{2}\left(k,t\right)=\frac{b_{z}\left(k,t\right)}{D_{0}Z_{0}},$$

where *D*_0_ denotes a dimensional normalization constant. By substituting Equation (28) in the relevant Maxwell’s curl equations, we then derive two coupled ordinary differential equations

(29) $$\begin{array}{ll} \frac{du_{1}}{dt} & =-icku_{2},\\ \frac{du_{2}}{dt} & =-ick\frac{u_{1}}{\varepsilon \left(t\right)}, \end{array}$$

subject to the initial conditions at *t* = *t*_0_

(30) $$u_{1}\left(k,t_{0}\right)=\frac{d_{y}^{\left(in\right)}\left(k,t_{0}\right)}{D_{0}},\;\;u_{2}\left(k,t_{0}\right)=\frac{d_{y}^{\left(in\right)}\left(k,t_{0}\right)}{D_{0}n_{1}},$$

where $d_{y}^{\left(in\right)}\left(k,t_{0}\right)$ denotes the plane-wave spectrum of the incident electric induction field at *t* = *t*_0_. For numerically solving Equation (29), we utilize the NDSolve routine available in Mathematica™ [38], which applies adaptively several numerical algorithms ranging from Runge–Kutta to implicit backward differentiation. In our implementation, we utilize default settings and parameters. Moreover, in order to favor numerical convergence, we implement the abrupt permittivity changes via an analytical, smooth unit-step function $U_{s}\left(t\right)=\left[\tanh\left(t/T_{s}\right)+1\right]/2$ , where *T*_*s*_ = *T*/100. From the numerical solution of Equation (29), by varying the momentum *k*, we finally synthesize the electric induction via Equation (27a) (with *d*_*y*_ = *D*_0_*u*_1_), numerically implemented via fast-Fourier-transform by means of the Fourier routine available in Mathematica™ [38].

Specifically, in Figure 2, we assume as a finite-energy source a windowed sinusoidal plane-wave,

(31) $$\begin{array}{ll} d_{y}^{\left(in\right)}\left(x,t\right) & =\sin\left[\frac{2\pi }{T}\left(t-\frac{x}{c}\right)\right]\left[U_{s}\left(t+20T-\frac{x}{c}\right)\right.\left.-U_{s}\left(t-\frac{x}{c}\right)\right], \end{array}$$

of duration 20*T*. Moreover, we assume the temporal variations of the refractive index starting at *t* = 0, and we sample the temporal evolution of the electric induction at a fixed location *x* = 10*λ*. Note that, since the Herpin-equivalent index *n*_*e*_ does not describe a physical material with a physical dispersion over a range of frequencies, but rather a mathematical equivalence that is strictly valid at a single frequency, we do not assume a physical dispersive model even in the case where *n*_*e*_ < 1 [Figure 2(C) and (D)], as our original intention is to demonstrate the Herpin equivalence for a single frequency. In our specific example, given the narrowband character of the chosen excitation, this does not lead to numerical-convergence problems.
